# Association of white matter hyperintensities with migraine features and prognosis

**DOI:** 10.1186/s12883-018-1096-2

**Published:** 2018-07-02

**Authors:** Hui Xie, Qiang Zhang, Kang Huo, Rui Liu, Zhi-Jie Jian, Yi-Tong Bian, Guo-Liang Li, Dan Zhu, Li-Hui Zhang, Jian Yang, Guo-Gang Luo

**Affiliations:** 1 Department of Neurology, The First Affiliated Hospital of Xi’an Jiaotong University, No. 277 Yanta West Road, Xi’an, 710061 Shaanxi China; 2 Department of Neurology, Shaanxi Provincial People’s Hospital, Xi’an, 710068 China; 3 Department of Radiology, The First Affiliated Hospital of Xi’an Jiaotong University, Xi’an, 710061 China; 4 Arrhythmia Unit, Department of Cardiovascular Medicine, The First Affiliated Hospital of Xi’an Jiaotong University, Xi’an, 710061 China

**Keywords:** Migraine, White matter hyperintensities, Clinical significance, Prognosis

## Abstract

**Background:**

White matter hyperintensities (WMHs) are frequently detected in migraine patients. However, their significance and correlation to migraine disease burden remain unclear. This study aims to examine the correlation of WMHs with migraine features and explore the relationship between WMHs and migraine prognosis.

**Methods:**

A total of 69 migraineurs underwent MRI scans to evaluate WMHs. Migraine features were compared between patients with and without WMHs. After an average follow-up period of 3 years, these patients were divided into two groups, according to the reduction of headache frequency: improved and non-improved groups. The percentage and degree of WMHs were compared between these two groups.

**Results:**

A total of 24 patients (34.8%) had WMHs. Patients with WMHs were significantly older (39.0 ± 7.9 vs. 30.6 ± 10.4 years, *P* < 0.001) and had a longer disease duration (median: 180.0 vs. 84.0 months, *P* = 0.013). Furthermore, 33 patients completed the follow up period (15 patients improved and 18 patients did not improve). Patients in the non-improved group had a higher frequency of WMHs (55.6% vs. 13.3%, *P* = 0.027) and median WMHs score (1.0 vs. 0.0, *P* = 0.030).

**Conclusions:**

WMHs can predict unfavorable migraine prognosis. Furthermore, WMHs may have a closer association with age than migraine features.

## Background

Migraine is a chronic debilitating headache characterized by recurrent moderate-to-severe headache attacks and autonomic nervous system related-symptoms [1]. Globally, migraine affects approximately 15% of the general population, and it preferentially affects females [2]. Migraine can be regarded as a risk factor associated with white matter hyperintensities (WMHs) [3], which are hyper-intense brain lesions in T2-weighted and Fluid-Attenuated Inversion Recovery (FLAIR) images [4]. Accumulating evidence documented the high incidence of WMHs in patients with migraine [3, 5]. However, the exact correlation between WMHs and the clinical features of migraine remain unclear. A population-based CAMERA study suggested the increased risk of WMHs in migraine patients with higher attack frequencies (≥1 attack per month), compared with patients with lower attack frequencies (< 1 attack per month) [3]. Trauninger et al. demonstrated that both disease duration and attack frequency were associated with WMHs in migraine patients [6]. On the other hand, Toghae et al. observed an association between WMHs and the age and migraine duration of patients, but not with attack frequency [7]. Recent studies have reported that WMHs are associated with the age of patients, and not with disease burden (disease duration and attack frequency) [8, 9]. Therefore, additional studies are required to gain insight into the exact correlation between WMHs and the features of migraine.

Larger or confluent WMHs are usually observed in cerebrovascular diseases and cognitive decline cases [10, 11]. However, WMHs associated with migraine tend to be punctate and mild [10]. Nevertheless, studies that have investigated the clinical implication of WMHs are scarce. Longitudinal population-based studies have previously indicated that WMHs in migraine are not associated with stroke or the decline in cognitive function [10, 12]. However, migraine has a variable short- or long-term prognosis. Some patients achieve complete or partial remission, while others experience persistent or even progressive attacks [13]. Recently, Eggers proposed that WMHs might be caused by multiple microemboli, which are induced by platelet aggregation abnormalities usually observed in migraine patients [14]. This supports the notion that WMHs may reflect an abnormal internal environment in patients. Therefore, the present study aimed to investigate the prognostic value of WMHs in migraine patients. To this end, the T1- and T2-weighted, as well as the T2-weighted FLAIR, magnetic resonance imaging (MRI) data obtained from migraine patients were analyzed to describe the imaging characteristics of WMHs. Furthermore, the association between WMHs and the clinical features of migraine were investigated, and the relationship between WMHs and migraine prognosis were examined.

## Methods

### Patients

A total of 69 migraine patients (52 females and 17 males, average age: 33.6 years old) were consecutively recruited from the Headache Clinic of the Department of Neurology, The First Affiliated Hospital of Xi’an Jiaotong University from February 2012 to November 2016. Inclusion criteria included: (1) patients with migraine who fulfilled the International Classification of Headache disorders (ICHD)-3 (β) criteria [1]), and (2) age between 12 and 55 years old. Exclusion criteria: (1) patients with major neurological diseases, (2) patients with major systemic diseases, (3) patients with thyroid diseases, (4) pregnant and/or lactating patients, and (5) patients with claustrophobia. The present study was approved by the Ethics Committee of the First Affiliated Hospital of Xi’an Jiaotong University (XJTU1AF2015 LSK-159), and an informed written consent was obtained from each patient. For minor patients (< 16), the written consent form was obtained from the accompanying parents.

### Demographic characteristics and clinical features

All enrolled patients were required to complete a standard questionnaire to collect basic clinical information at the Headache Clinic. This questionnaire assessed the demographic characteristics, past history, family history and features of migraine, and any accompanying symptoms and self-rating depression scale (SDS). Migraine features include disease duration, attack frequency, attack duration and the visual analogue scale (VAS) score. In addition, the questionnaire also investigated the history of smoking, hypertension, diabetes, oral contraceptive use, past vascular events, heart diseases, tumors, intracranial organic diseases and other medical conditions.

### Image data acquisition and evaluation of WMHs

All participants underwent MRI scans at the Department of Radiology, The First Affiliated Hospital of Xi’an Jiaotong University, using a 3.0 T GE Discovery MR scanner and a standard 8-channel phase array head coil. High-resolution structural images were acquired using a three-dimensional T1-weighted sequence with the following parameters: TR, 10.276 ms; TE, 4.9 ms; matrix, 256 × 256; section-thickness, 1 mm; FOV, 256 mm. T2-weighted images were acquired with the following parameters: TR, 6000 ms; TE, 104.4 ms; matrix, 385 × 384; section-thickness, 5 mm; FOV, 240 mm. FLAIR images were acquired with the following parameters: TR, 9102 ms; TE, 168.7 ms; matrix, 288 × 224; section-thickness, 5 mm; FOV, 240 mm.

WMHs were visible as hyperintense lesions on FLAIR images, and as isointense or slightly hypointense lesions on T1-weighted images. MRI scans were assessed for the number and features of WMHs, including the appearance, number, size and anatomical location. All MRI scans were reviewed by an experienced neurologist and neuroradiologist. The degree of WMHs was assessed using the Scheltens visual rating scale [15]. Briefly, WMHs were separately graded in each of the following locations: frontal lobes, temporal lobes, parietal lobes and occipital lobes. WMHs were graded as follows: 0 (no lesions), 1 (hyperintensity < 3 mm and *n* ≤ 5), 2 (hyperintensity < 3 mm and *n* ≥ 6), 3 (hyperintensity 4–10 mm and *n* ≤ 5), 4 (hyperintensity 4–10 mm and *n* ≥ 6), 5 (hyperintensity ≥11 mm and *n* ≥ 1), and 6 (confluent). The sum of scores from each location was considered as the final score [15, 16]. According to WMH, migraine patients were divided into two groups: non-WMH group (complete absence of WMHs or WMHs score = 0) and WMH group (presence of WMHs or WMHs score ≥ 1). Next, the features of WMHs and its correlation to the clinical variables were analyzed. Finally, a retrospective follow-up study was conducted to analyze the association of WMHs and migraine prognosis.

### Patient follow-up

**Fig. 1:**
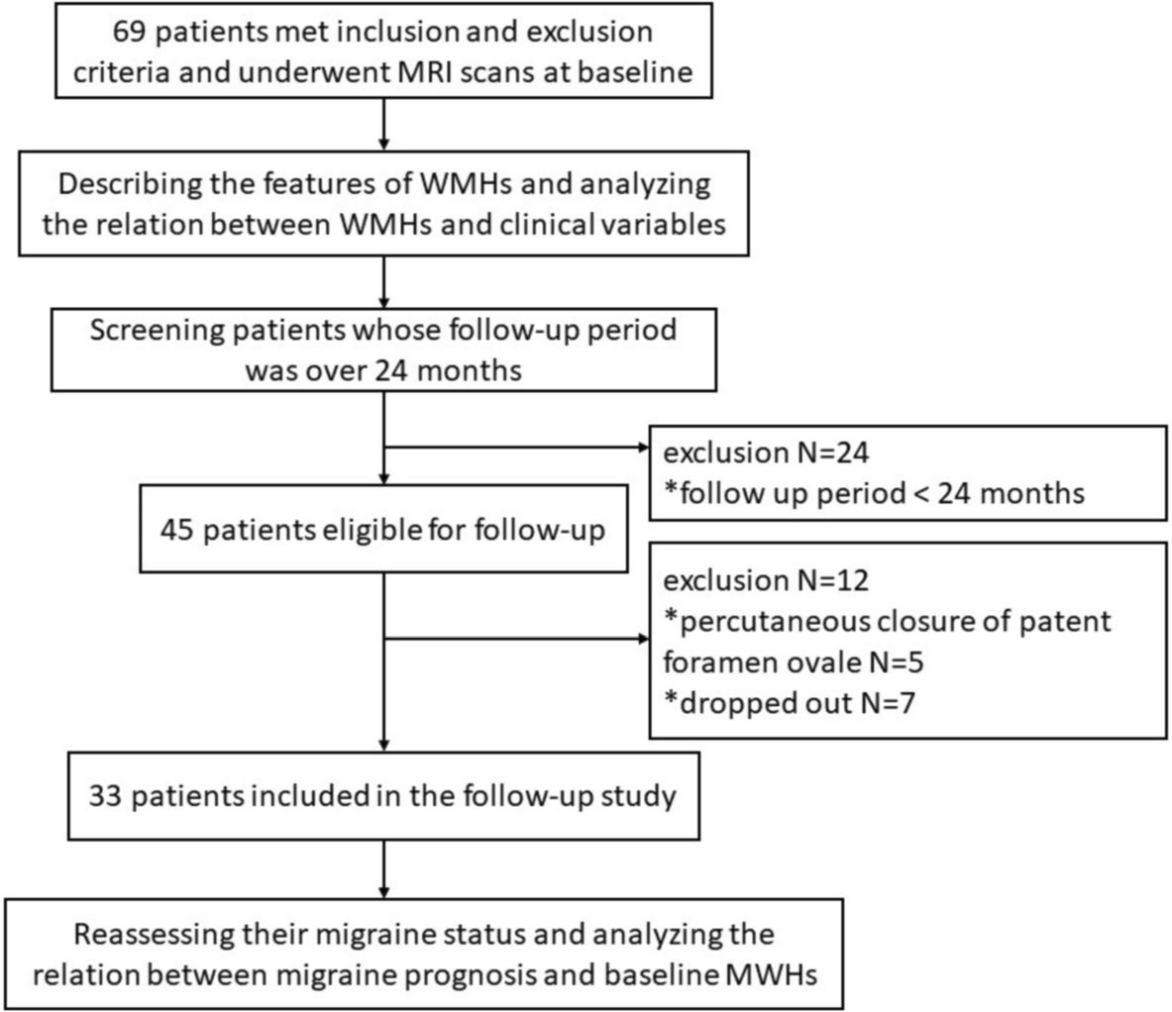
A flow chart demonstrating the study design

In December 2016, patients who were enrolled in the study for a mean period of > 24 months (*n* = 45) were recruited for a follow up visit. A total of five patients were excluded due to percutaneous closure of the patent foramen ovale, and another seven patients dropped out from the follow up study. The study flow chart is illustrated in Fig. 1.

A total of 33 patients were re-interviewed to assess their migraine status and determine the present headache attack frequency per month. Patients were divided into improved and non-improved groups based on the mean percentage of attack frequency calculated from the 3 months that preceded the follow-up appointment. The outcome was defined as improved if the attack frequency decreased by more than 50% compared to that of baseline. On the other hand, if the attack frequency failed to decrease by more than 50% at follow up, the patient was considered to be non-improved. Next, the outcome was further classified into four categories including complete remission, partial remission, persistence and progression. Complete remission was defined as zero migraine attacks in the 3 months that preceded the follow-up. Partial remission was defined as the reduction of migraine frequency by more than 50%. Persistence group had the change of migraine frequency hovering around the 50%. Progression was defined as the increase of migraine frequency by more than 50%. It is worth mentioning that despite our efforts, most of the patients who were followed up denied to undergo a follow-up MRI scan.

### Therapeutic regimen

The usage of medication during the follow-up period was recorded. Migraine patients were administered with migraine prophylactic medications (including calcium channel blockers, anticonvulsants, or β-blockers) or non-steroidal anti-inflammatory drugs, according to the recommendation of physicians.

### Statistical analysis

Demographic and clinical characteristics were tabulated using descriptive statistics, including percentages, quartiles (non-normal data) and means (normal data). Chi-square test and Fisher’s exact probability test were used to test for differences in categorical data. Student’s *t*-test was used to compare the means of normally distributed variables. Non-parametric tests were used for non-normally distributed data. A correlation analysis between WMHs and patient prognosis was performed using Spearman correlation. Further, logistic regression was conducted to evaluate contributing factors to migraine prognosis. Statistical analyses were performed using the SPSS 23.0 software. A *P*-value < 0.05 was considered statistically significant.

## Results

### Demographic characteristics and migraine features

A total of 69 patients with an average age 33.6 years old (range: 14–54 years old) were enrolled in the present study. Among these patients, 52 patients (75.4%) were females. The demographic characteristics and clinical features of migraine are summarized in Table 1. Furthermore, 19 patients (27.5%) presented with aura including visual aura (*n* = 16), visual and sensory aura (*n* = 2), and brainstem aura (*n* = 1). Five patients were presented with chronic migraine, while the remaining patients were episodic. Moreover, a total of eight patients (11.6%) were smokers, and another five patients (7.2%) were hypertensive, but none of the enrolled patients were diabetic.

**Table 1:** Comparison of clinical characteristics between the non-WMH group and WMH group

	Non-WMH group (*n* = 45)	WMH group (*n* = 24)	*P*-value
Age (year, mean ± SD)	30.6 ± 10.4	39.0 ± 7.9	< 0.001*
Gender			0.592
Female, *n* (%)	33 (73.3%)	19 (79.2%)	
Male, *n* (%)	12 (26.7%)	5 (20.8%)	
BMI (kg/m^2^, mean ± SD)	20.7 ± 2.9	21.8 ± 2.2	0.127
Hypertension, *n* (%)	2 (4.4%)	3 (12.5%)	0.458
Smoking, *n* (%)	5 (11.1%)	3 (12.5%)	1.000
Oral contraceptive use, n (%)	1 (2.9%)	1 (5.3%)	1.000
Headache characteristics			
Aura, *n* (%)	14 (31.1%)	5 (20.8%)	0.363
Disease duration (month, quartile)	84.0 (42.0, 198.0)	180.0 (75.0, 297.0)	0.013*
Attack frequency (day/month, quartile)	3.0 (2.0, 7.0)	4.0 (2.0, 10.0)	0.465
Attack duration (hour, quartile)	5.0 (3.0, 10.0)	9.5 (4.0, 24.0)	0.172
Visual analogue scale, mean ± SD	7.0 ± 1.9	7.8 ± 1.5	0.080
Accompany symptoms			
Nausea, *n* (%)	37 (84.1%)	21 (87.5%)	0.983
Vomiting, *n* (%)	28 (63.6%)	15 (62.5%)	0.926
Photophobia, *n* (%)	32 (72.7%)	18 (75.0%)	0.839
Phonophobia, *n* (%)	33 (75.0%)	18 (75.0%)	1.000
Dizziness, *n* (%)	21 (47.7%)	13 (54.2%)	0.612
Family history of migraine, *n* (%)	26 (57.8%)	11 (45.8%)	0.343
SDS scores (quartile)	42 (36, 56)	44.5 (37.3, 56.8)	0.653

*A significance level, *P* < 0.05; *SD* standard deviation, *BMI* body mass index

**Fig. 2:**
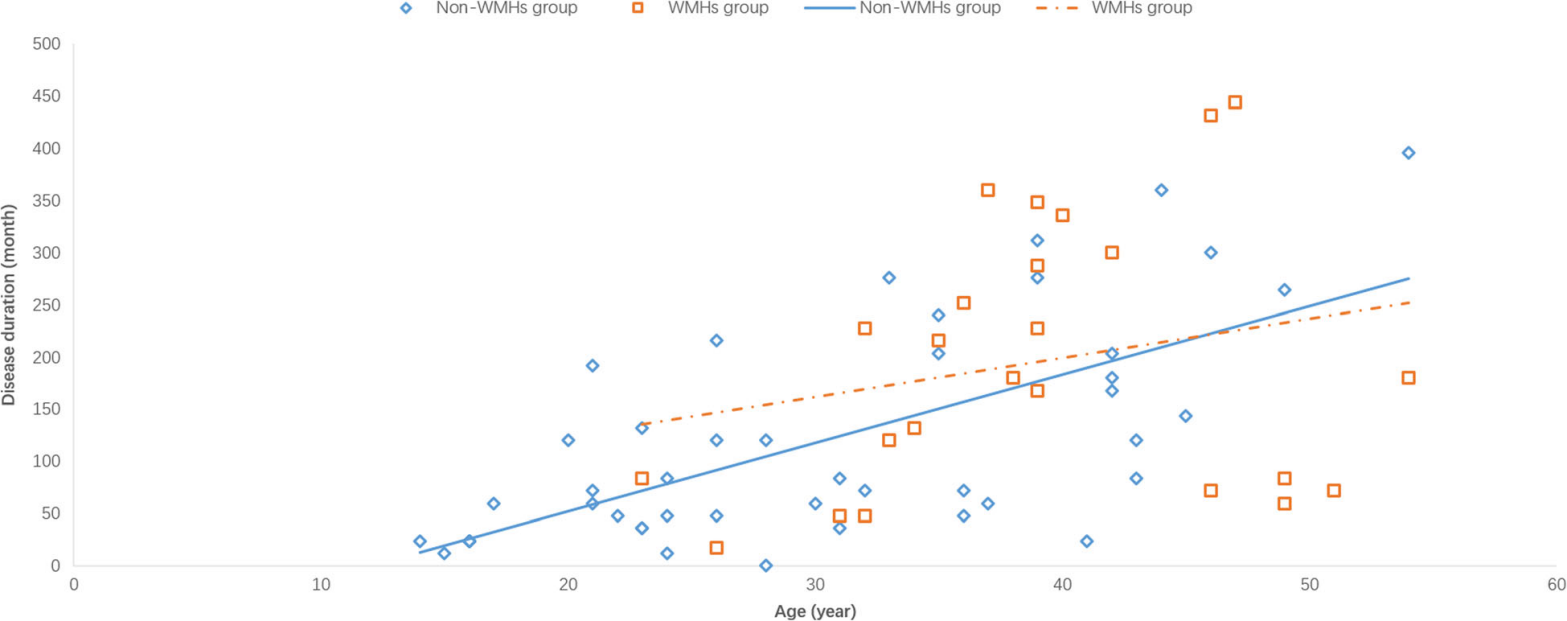
A scatter plot showing the changing trend of disease duration with age

Next, WMHs were investigated through T2-weighted and FLAIR MRI scans. According to WMH, migraine patients were divided into two groups: non-WMH group (complete absence of WMHs or WMH score = 0) and WMH group (the presence of WMHs or a WMH score of ≥1) (Table 1). Among these 69 migraine patients, a total of 24 patients (34.8%, 19 females and five males) presented with WMHs. Furthermore, there was a significant difference in age between the WMH and non-WMH groups. Patients in the WMH group were significantly older compared to patients in the non-WMH group (39.0 ± 7.9 years vs. 30.6 ± 10.4 years; *P* = 0.000). Among the disease burden related variables, disease duration was significantly higher in the WMH group than in the non-WMH group (median: 180 months vs. 84 months; *P* = 0.013). Furthermore, a moderate positive correlation was observed between age and disease duration (*r* = 0.589, *P* < 0.001), which indicate a possible confounding effect of age in the association between disease duration and WMHs. A scatter plot was preformed to show the changing trend of disease duration with age. The trend was similar between the improved group and non-improved group (Fig. 2). No significant difference was observed in the presence of aura between these two groups. It is worth mentioning that the exclusion of hypertension or smoking status (12 cases) did not affect the data analyses between these two groups.

### Features of WMHs

**Fig. 3:**
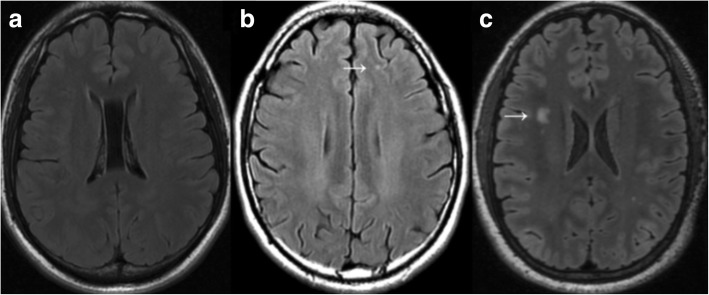
Representative axial FLAIR images of WMHs: (**a**) Normal brain structures without white matter hyperintensity. **b** A punctate hyperintense lesion (arrow) in the right frontal lobe. **c** A confluent lesion (arrow) and some punctate lesions in the brain

**Fig. 4:**
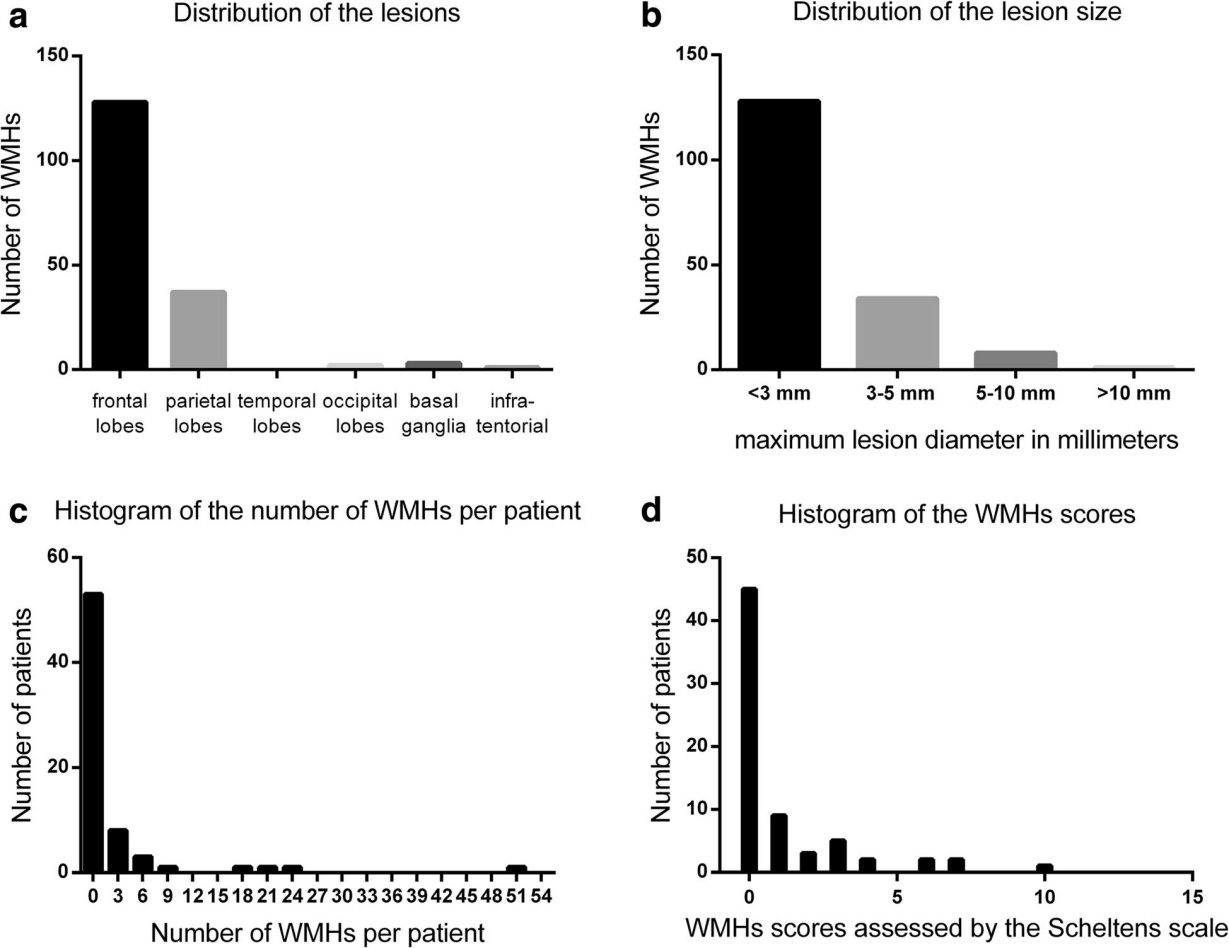
(**a-d**) Bar charts characterizing the imaging features of WMHs in migraine patients: (**a**) a bar chart representing the anatomical distribution of WMHs, (**b**) a bar chart representing the recorded sizes of WMHs in migraine patients, (**c**) a bar chart representing the distribution of the number of WMHs per patient, and (**d**) a bar chart representing the distribution of WMHs scores

In the WMH group (*n* = 24), most lesions were punctuate (22 patients), and two patients presented with confluent lesions (Fig. 3b and c). Among patients with WMHs, a total of 171 lesions were detected. WMHs were significantly higher in the frontal lobes (74.9%), followed by the parietal lobes (21.6%) (Fig. 4a). Furthermore, it was observed that WMHs in migraine patients were generally mild, most lesions (94.7%) were < 5 mm (Fig. 4b), and the average number of lesions per patient was generally small, with a median number of 2.5 (range: 1–52) (Fig. 4c). According to the Scheltens scale, the WMH scores of patients were low, with a median score of 2.5 (range: 0–10) (Fig. 4d). Furthermore, the difference in numbers and scores between males and females with WMHs was not statistically significant.

### Correlation between WMHs and migraine prognosis

A total of 33 patients were followed up in the present study (Fig. 1). The average follow-up period was 3 years, which ranged within 2–4 years. Among these 33 re-assessed patients, 15 patients (45.5%) were assigned to the improved group while 18 patients (54.5%) were assigned to the non-improved group. Specifically, 4 patients (12.1%) achieved complete remission and they were free of migraine attacks for more than 1 year. Eleven patients (33.3%) achieved partial remission. Fourteen patients (42.4%) were in persistence group. Four patients (12.1%) were in the progression group. The four patients attaining complete remission were 30, 34, 39 and 41 years old at follow-up, excluding the potential effect of menopausal state. Differences in age, gender, or BMI between the improved and non-improved groups were not statistically significant. None of our patients had hypertension or diabetes. Among the headache characteristics, aura was more frequent in the improved group (60.0% vs. 11.1%, *P* = 0.008) (Table 2).

**Table 2:** Comparison of demographics and WMHs between the improved and non-improved groups

	Improved group (*n* = 15)	Non-improved group (*n* = 18)	*P*-value
Age (year, mean ± SD)	29.5 ± 9.8	34.7 ± 11.0	0.166
Gender			1.000
Female, *n* (%)	13 (86.7%)	16 (88.9%)	
Male, *n* (%)	2 (13.3%)	2 (11.1%)	
BMI (kg/m^2^, mean ± SD)	19.9 ± 2.3	21.1 ± 2.9	0.203
Headache characteristics			
Aura, *n* (%)	9 (60.0%)	2 (11.1%)	0.008*
Disease duration (month, quartile)	72 (24, 144)	150 (48, 279)	0.096
Attack frequency (day/month, quartile)	4.0 (2.0, 7.0)	2.0 (2.0, 5.5)	0.555
Attack duration (hour, quartile)	4.5 (2.0, 10.0)	11.0 (3.4, 24.0)	0.204
Visual analogue scale, mean ± SD	7.0 (6.0, 7.9)	8.0 (7.0, 9.3)	0.051
Medications			
Prophylactic medications^#^, *n* (%)	5 (33.3%)	4 (22.2%)	0.697
Medication overuse^#^, *n* (%)	1 (6.7%)	2 (11.1%)	1.000

*A significance level, *P* < 0.05; ^#^The number of patients who took regular prophylactic medications for more than 3 months or had medication overuse during the follow-up period; *SD* standard deviation, *BMI* body mass index

Among the 18 patients in the non-improved group, 10 patients had one or more WMHs, while WMHs were detected in only two of 15 patients in the improved group, and the difference was statistically significant (*P* = 0.027, Table 3). Furthermore, patients in the non-improved group had a significantly higher median WMHs score compared to patients in the improved group (*P* = 0.030).

**Table 3:** Fisher’s exact test representing the correlation between WMHs and patient prognosis

	Improved	Non-improved	Total
WMHs group	2	10	12
Non-WMHs group	13	8	21
Total	15	18	33

Fisher’s exact test: two-tailed *P*-value = 0.027

Next, we examined the impact of prophylactic treatments on the migraine outcome. We compared the rate of regular prophylactic treatment between the improved and non-improved groups administered for more than 3 months regardless of drug classes or doses. Our results demonstrated the absence of significant difference between both patient groups (Table 2). It is worth mentioning that none of the patients reported the use triptans.

Multivariate logistic regression analysis was conducted to analyze the independent risk factor associated with the non-improved outcome. The impact of age, aura, disease duration, VAS and the presence of WMHs were examined. Our results showed that WMHs (OR = 12.6, 95% CI (1.093~ 145.848) and aura (OR = 0.04, 95% CI (0.002 ~ 0.683) were the independent risk factors associated with the non-improved outcome.

In the follow up group, patients without aura were significantly older than those with aura (36.6 ± 9.1 vs. 23.8 ± 8.3, *P* < 0.001) and had a significantly longer disease duration (median: 162 months vs. 36 months, *P* = 0.003) and attack duration (median: 2 h vs. 11 h, P < 0.001). Patients without aura also had a higher incidence of WMHs than those with aura (45.4% vs. 18.2%, *P* = 0.249). The difference was obvious although it did not achieve the statistical significance.

## Discussion

Migraine is a well-documented risk factor for WMHs [3, 5, 17, 18]. However, to date, the clinical significance of WMHs in migraine prognosis remains unclear. Therefore, in the present study, we explored the association between WMHs and migraine prognosis. Results demonstrated that the presence and degree of WMHs can be associated with unfavorable migraine prognosis. In addition, in the present study, we described the features of WMHs in migraine patients, and observed that WMHs were positively correlated with old age.

WMHs are commonly associated with physiological conditions such as aging and pathological conditions associated with vascular risks such as hypertension [19]. Pathologically, WMHs can result from local brain ischemia at the microvascular level [14, 19]. Several reports have demonstrated a higher incidence of WMHs in patients with migraine, compared to healthy control subjects [3, 5]. In the same context, Schurks et al. demonstrated that migraine is a definite risk of stroke, especially in young women [20]. This suggests the role of ischemia in the mechanism of WMHs in association with migraine. To date, the exact pathophysiology of WMHs is not well-understood. Accumulating evidence revealed that migraine patients may have abnormal platelet activation, impaired endothelial function and hypercoagulability [21–23], which can be potential causes for the development of WMHs. These abnormal vascular conditions might favor the persistence or even the progression of migraine. Consequently, it is reasonable to speculate that WMHs can be correlated with unfavorable migraine prognosis. Indeed, results obtained from the present study demonstrate that both the degree and frequency of WMHs were positively correlated with unfavorable migraine prognosis. To the best of our knowledge, this is the first report that demonstrated the prognostic value of WMHs in migraine patients.

Aging is another important risk factor of the development of WMHs [4, 19]. Among the examined clinical variables, only age and disease duration were correlated to WMHs in our patient cohort. However, a moderate positive correlation was observed between age and disease duration. The disease duration of patients with WMHs was not higher than that of patients without WMHs within the same age brackets. These results imply that the association between disease duration and WMHs could result from the confounding effect of age. Furthermore, we investigated whether age had a confounding effect on the association of WMHs and migraine prognosis. There was no significant difference in age between the improved and non-improved groups. Regression analysis showed WMHs was the independent risk factor for the non-improved outcome with the control of the cofounding effect of age. Finally, the rate of prophylactic treatment was also comparable between these two groups. Collectively, these results indicate that the association between WMHs and migraine prognosis were not affected by age or medications in our patient cohort. However, it is recognized that the prevalence of migraine increases with age from childhood to adulthood, and it peaks at 35 to 39 years of age, after which it gradually decreases, particularly among women after menopause [3]. Meanwhile, WMHs are not static, and in most cases WMHs progress with aging [24, 25]. Therefore, for older migraine patients, WMHs will not serve as a reliable marker for prognosis. It is worth mentioning that a 3-year longitudinal follow-up study revealed a non-significant increase in the number of WMHs in 19.5% of the patient cohort [26]. These results suggest that WMHs would not significantly progress within a relatively short period (3 years). In the present study, patients were re-evaluated after 2–4 years (mean: 3 years). Therefore, this might indicate that the validity of WMHs in migraine prognosis is at least applicable over a relatively short interval (3-year window). Future research should examine its validity through long-term follow ups.

In this study, our results demonstrated that migraineurs with favorable outcome had a higher incidence of aura. Studies analyzing the relationship between the presence of aura and migraine prognosis are scarce. Dahlof et al. observed that aura was associated with poor migraine prognosis in females, but a similar relationship was not observed in male patients [27]. A 5-year follow-up study that investigated the outcome of migraine in children and adolescents failed to find a significant difference between migraine with and without aura, although the percentage of subjects who were free from migraine at follow-up was 30.6% in the case of migraine with aura and 20.3% in the case of migraine without aura [28]. In our study cohort, patients with aura were significantly younger than those without aura. Disease duration and attack duration were significantly lower in patients with aura than those without aura. Patients with aura had a lower incidence of WMHs than those without aura although the difference was not significant. On the other hand, Gozke et al. previously suggested a higher incidence of WMHs in migraine with aura [29]. Moreover, it is well recognized that migraine with aura is a risk associated with ischemic stroke [20]. Therefore, it is plausible to speculate that the better baseline headache condition in patients with aura could contribute to better prognosis compared to patients without aura. However, the exact reason remains to be clarified in future studies.

There was no significant difference of SDS scores between WMHs group and Non-WMHs group. The relationship between depression and WMHs remains unclear [30, 31]. A meta-analysis showed a significant weak association between WMHs and depression (OR: 1.02~ 1.22) [32]. The burden of migraine can be assessed by disease duration, attack frequency, attack duration and headache intensity (VAS) [18]. In the present study, we investigated the impact of WMHs on disease burden. It was observed that WMHs were significantly associated with longer disease duration, while a significant correlation with attack frequency or its duration was not observed. To date, the association between WMHs and migraine features remain controversial [6, 18, 33, 34]. Earlier reports have consistently revealed that WMHs were not associated with migraine features, including disease duration or attack duration [35–37]. On the other hand, Gozke et al. demonstrated that WMHs were associated with a higher frequency of longer disease duration and higher attack frequencies [29]. Similarly, the CAMERA study supported the same conclusions [3]. However, the population of the CAMERA study had high proportions of vascular risk factors (32–42% prevalence of hypertension, 60–66% prevalence of smoking, and 19–29% prevalence of oral contraceptive use), which may lead to confounding bias. This discrepancy might be attributed to the accuracy of the MRI techniques, especially the blurring artifact. Other factors include the demographic characteristics of the study population or discrepancies in the study design [3, 6, 29, 34, 35]. In addition, the measurements of disease burden are usually not stable; that is, disease duration was always affected by age, and attack frequency and attack duration often show changeable patterns over time; while the VAS had strong subjectivity. Therefore, future research should focus on more stable parameters to assess the disease burden of migraine.

Taken together, the results obtained from the study suggest that WMHs may predict unfavorable migraine prognosis. Therefore, our results could lead to the alteration of the treatment protocol for migraineurs with WMHs. That is, physicians could apply more positive treatment strategies to achieve a more favorable prognosis in patients with high WMHs scores. Furthermore, our results also indicate that WMHs have a closer association with age than the clinical features of migraine.

Nevertheless, the present study had a few limitations. The relatively small number of enrolled patients is considered to be the main limitation. Furthermore, the absence of a control group precluded definitive conclusions about the nature of the observed alterations in WMHs or whether their degree is beyond normal aging. Age should be controlled in the design of the study. Thus, future work should focus on investigating the implication of WMHs among relatively young migraine patients. Similarly, the heterogeneity of the patient cohort such as migraine with and without aura, episodic migraine and chronic migraine, should be improved. Different migraine types possibly have different effects on the prognosis. The relatively low migraine frequency at baseline in our study is also a major limitation as prognostic information may be of greater value in high frequency migraine states. However, the small sample size limited the stratified analysis of frequency in the case of controlling the effect of age. The method for migraine prognosis categorization was one-sided. It reflected the change of frequency but it did not investigate the current frequency level, headache intensity or even response to acute therapy. However, there is no standard prognostic categorization for migraine yet. Future prospective multicenter studies with more controlled conditions (migraine type and age) and long-term follow up should be conducted to confirm these results. Future studies should also employ more stable parameters that assess disease burden, in order to further confirm the clinical significance of WMHs. It is worth mentioning that in the present study, we could not definitively investigate other WMH-associated risk factors, including hypertension, diabetes, hyperlipidemia, hyperhomocysteinemia, hyperuricemia, hypercoagulability, heart diseases, kidney diseases, inflammation and autoimmune diseases. These conditions may impact the strength of our conclusions regarding the nature of WMHs and their effect on migraine. Future studies should be controlled for the confounding effects of the above mentioned conditions.

## Conclusion

This study suggests that WMHs can predict short-term unfavorable migraine prognosis, thereby providing a new insight into the clinical significance of WMHs in migraine. Meanwhile, it demonstrates that WMHs in migraine patients are generally mild, mostly located in the frontal and parietal lobes, and may have a closer association with age than headache characteristics.

## Abbreviations

FLAIR: Fluid-attenuated inversion recovery
VAS: Visual analogue scale
WMHs: White matter hyperintensities
